# Strategies to improve recruitment in mental health clinical trials: a scoping review (RE-MIND study)

**DOI:** 10.1186/s13063-024-08665-x

**Published:** 2024-12-18

**Authors:** Mais Iflaifel, Charlotte L. Hall, Heidi R. Green, Andrew Willis, Stefan Rennick-Egglestone, Edmund Juszczak, Mark Townsend, Jennifer Martin, Kirsty Sprange

**Affiliations:** 1 Nottingham Clinical Trials Unit, University of Nottingham, Nottingham, UK; 2 NIHR MindTech MedTech Co-operative, Institute of Mental Health, School of Medicine, Mental Health and Clinical Neurosciences, University of Nottingham, Innovation Park, Triumph Road, Nottingham, UK; 3 NIHR Nottingham Biomedical Research Centre, Institute of Mental Health, University of Nottingham, Innovation Park, Triumph Road, Nottingham, UK; 4 Health Services Research Unit, University of Aberdeen, Aberdeen, UK; 5 COUCH Health, Manchester, UK; 6 Centre for Ethnic Health Research, Leicester/Diabetes Research Centre, University of Leicester, Leicester, UK; 7 School of Public Health, University College Cork, Cork, Ireland; 8 HRB Clinical Research Facility, University College 8 Cork, Cork, Ireland; 9 School of Health Sciences, Institute of Mental Health, University of Nottingham, Nottingham, UK; 10 NIHR Evaluation, Trials and Studies Coordinating Centre (NETSCC), Southampton, UK

**Keywords:** Mental health, Recruitment, Randomised controlled trials, Evidence review, Diversity, Inclusivity

## Abstract

**Background:**

Lower-than-expected recruitment continues to be one of the major causes of trial delays, and trials to improve mental health are no exception. Indeed, recruitment challenges in trials of vulnerable populations, such as those living with mental health illness, can even be exacerbated. To address this, researchers are turning to digital and online recruitment strategies, e.g. web-based approaches and multi-media in order to (1) increase recruitment efficiency (recruit to target and on time) and (2) improve diversity in mental health clinical trials to be more inclusive and reduce health inequity. There is, however, inconclusive evidence on the success of digital and online recruitment strategies in mental health clinical trials. The RE-MIND study comprised a scoping review to assess the impact of using such recruitment strategies in mental health clinical trials to inform a more systematic scoping review.

**Methods:**

A cohort of 191 recently published RCTs and randomised feasibility studies were identified from the NIHR Journals Library and top two mental health journals (based on citation metrics), Lancet Psychiatry and JAMA Psychiatry. Population characteristics including gender, ethnicity and age were summarised for inclusivity using descriptive statistics, and recruitment strategies were compared to examine differences in their success in recruiting to target.

**Results:**

After screening, 97 articles were included for review. The review findings showed no evidence that offline or mixed strategies were superior for achieving recruitment targets in mental health trials. However, there was a suggestion that trials using a mixed recruitment strategy improved inclusivity and tended to recruit closer to the target.

**Conclusions:**

The key finding was that consideration should be given to a mixed methods approach to recruitment not only to enable wider and more diverse participation in mental health trials but also to realize greater efficiency.

**Supplementary Information:**

The online version contains supplementary material available at 10.1186/s13063-024-08665-x.

## Background

Randomised controlled trials are required to generate evidence for efficacy/effectiveness and safety of interventions. Recruitment is a critical part of the clinical trial process, but it is also challenging, as trials often fail to meet their target sample size due to delays in recruitment [1]. Research investigators often overestimate the pool of available participants who meet the inclusion criteria or would be willing to participate in a clinical trial [2]. This ultimately results in slow and/or insufficient recruitment and unrepresentative samples that can lead to underpowered trials, uncertain results and poor implementation [3].

Traditionally, recruitment for clinical trials, and for mental health research specifically, has been critically dependent upon face-to-face referrals or contacts. However, there is growing recognition of the need to utilise a number of different and more effective recruitment strategies tailored to the study design and population [4]. Hence, there has been an increasing switch to online recruitment strategies such as web-based approaches and multi-media to recruit participants for mental health research [5]. In comparison to offline strategies, online strategies may enable wider reach and be more cost-effective to target specific populations to meet recruitment goals and, in some cases, reduce recruitment time [5–7]. In addition, online strategies may also reach communities that are not currently under the care of specialist mental health services. This may be particularly important for conditions where specialist mental health care is only offered at centres typically in large cities or when widening recruitment to minority communities.

A recent study looked at data from NIHR HTA trials of mental health and found that 60% failed to reach their original recruitment target. The authors reflected upon how online recruitment and consent may navigate some of these issues [8]; however, they did not conduct a formal comparison between online- and offline-conducted trials. Brogger-Mikkelsen et al. [9] found that 12/23 (52%) of studies that used an online recruitment strategy had a better recruitment rate when compared to offline recruitment strategies. However, it is not clear what demographic characteristics may influence this, and the number of papers examined was limited due to the authors’ inclusion criteria.

There is evidence of a number of barriers to recruit people with mental health issues into mental health clinical trials, such as distrust and suspicion of researchers [10, 11], concerns about confidentiality [12] and the stigma compounded by feelings of mistrust and scepticism of mental health research [13]. Recent research by Spanakis et al. also demonstrated that those living with severe mental ill health may not have the necessary digital skills to engage with online research [14]. In addition, the issue of poor ethnic diversity in mental health trials in the UK and globally is particularly concerning, given that people from ethnic minority backgrounds experience disproportionately high levels of adverse mental health [15]. It is important that the characteristics of trial participants are reflective of the population of interest to ensure generalisability of the results [16].

In response to the ongoing need to reduce health care inequality, partners across the clinical trial ecosystem have increased their efforts to enhance diversity in clinical trials. For example, the UK National Institute for Health and Care Research (NIHR) Clinical Research Network commissioned the INCLUDE project [17] have developed the Equality, Diversity and Inclusion (EDI) Strategy 2022–2027 [18] to provide a framework to ensure the implementation of inclusive practice in research, culture and systems. The Food and Drug Administration (FDA) also issued guidance on enhancing the diversity of clinical trial populations [19].

A survey of 367 adults with a severe mental health diagnosis found that approximately 30% of patients with psychosis reported their use of the internet during the pandemic as ‘a lot’ [20]. Yet despite this, there are considerable concerns around the use of online strategies to recruit people with mental health issues, such as the digital divide—defined as the gap between people who have access to the Internet and those who do not or who have restricted access [14]. Other concerns include low technology skills [21], apps/websites/interfaces that are not user-friendly, concerns around data security and the ability to differentiate between trustworthy messages and spam plus fraudulent misinformation on the Internet [22–24].

Existing research into online recruitment has focused on the attainment of overall trial recruitment targets and perceived barriers rather than participant characteristics [8]. There is contradictory evidence regarding trials using online recruitment methods. While some trials have reported particularly good recruitment [25], other evidence from a systematic review indicated that online trials may be particularly susceptible to poor recruitment and limited engagement with the intervention [24].

### Aims and objectives

This scoping review is part of a wider project, REcruitment in Mental health trials: broadening the ‘net’, opportunities for INclusivity through online methoDs (RE-MIND). The RE-MIND study protocol is available from the Nottingham Clinical Trials Unit (NCTU) website [26]. RE-MIND aimed to identify and provide considerations for the use of online methods in the recruitment of participants to mental health RCTs, with a focus on whether online methods can enhance inclusivity. This review is a preliminary study designed to gather data to inform the design of a more systematic review in which a wider search strategy can be employed. The qualitative findings [27] and our recommendations for future research have previously been published [28].

The main objective was to determine the proportion of trials/studies using online recruitment strategies that recruited to target and assess whether these strategies were associated with a more diverse participant population in terms of gender, age and ethnicity.

## Methods

### Design

A scoping review of recently published randomised controlled trials (RCTs) and randomised feasibility or pilot studies in mental health to gather data on and assess the impact of online recruitment compared to offline recruitment.

Data sources were the NIHR Journals Library and two top mental health journals (based on citation metrics), Lancet Psychiatry and JAMA Psychiatry, which were searched for RCTs that met our inclusion criteria. This cohort was designated as representing high-quality research in the field of mental health. Filters were set to select the most recent evidence using the latest online recruitment strategies. Three reviewers individually conducted screening articles (MI, KS and CLH) to confirm eligibility. Where there was uncertainty over eligibility, discordant cases were discussed and agreed upon by the three reviewers.

Inclusion criteria:

1. Published between 1 January 2017 and 30 June 2022.
2. Randomised controlled trials or randomised feasibility or pilot studies.
3. Studies using offline, online or both (mixed) strategies for participant recruitment/identification.
4. Studies delivered in a health care, community or secure setting (e.g. prisons/youth offending institutions).
5. Study population reached a pre-defined cutoff on a mental health scale (as defined by the trial authors or meeting a DSM or ICD criteria).
6. Interventions designed to improve mental health (as defined by the trial authors).

Exclusion criteria:

1. Studies of prevention or addressing addiction.
2. Articles with a main focus on other conditions (not mental health).
3. Articles on interventions for activities of daily living, self-care, independence or lifestyle (where the intervention did not directly treat the disorder).
4. Non-patient participant studies, e.g. health care providers.

### Data collection

The study team used the following definitions to guide data collection.

- Online recruitment strategies were defined as the use of internet technologies to recruit research participants such as social media advertisements, Google search engine advertisements and other website campaigns [9].
- Offline recruitment strategies were defined as in-clinic recruitment, soliciting potential participants through mail and telephone using health records and registers, media campaigns, newspaper advertisements and via radio and television talks [9].
- Mixed recruitment strategies were any combination of online and offline strategies used in the same study.

Data were extracted using a bespoke tool (developed by MI, KS and CLH) and reviewed by the multidisciplinary co-investigator study team (HG, AW, EJ, MT) focusing on:

- Trial design, publication year, type of intervention, diagnosis, setting and location by country.
- Whether the trial reached the planned sample size target.
- Type of recruitment strategies used.
- Textual data on efficiency of recruitment strategies used, as reported by the trial authors, if provided.
- Individual participant baseline characteristics (e.g. age, gender, ethnicity).

It is worth noting that the concepts of gender and sex are often incorrectly used interchangeably, and the reporting of what authors mean when they are talking about sex and/or gender is limited [29]. The articles included in this review identified male/female/other, e.g. non-binary which we have referred to as gender.

The data extraction tool was piloted on a sample of papers from all three sources. MI, KS and CLH then carried out the data extraction by dividing up the included papers.

### Analysis

The quantitative data extrapolated was guided by the NIHR INCLUDE list of under-served groups [17]. Descriptive data on the published trials that met the eligibility criteria was presented by the type of recruitment strategy used.

Study characteristics and outcomes were summarised with counts and percentages for categorical variables. Comparative analysis used the Fisher’s exact statistical test to explore whether there was an association between recruitment strategy (offline, mixed and online) and whether the trial recruited to target.

## Results

**Fig. 1:**
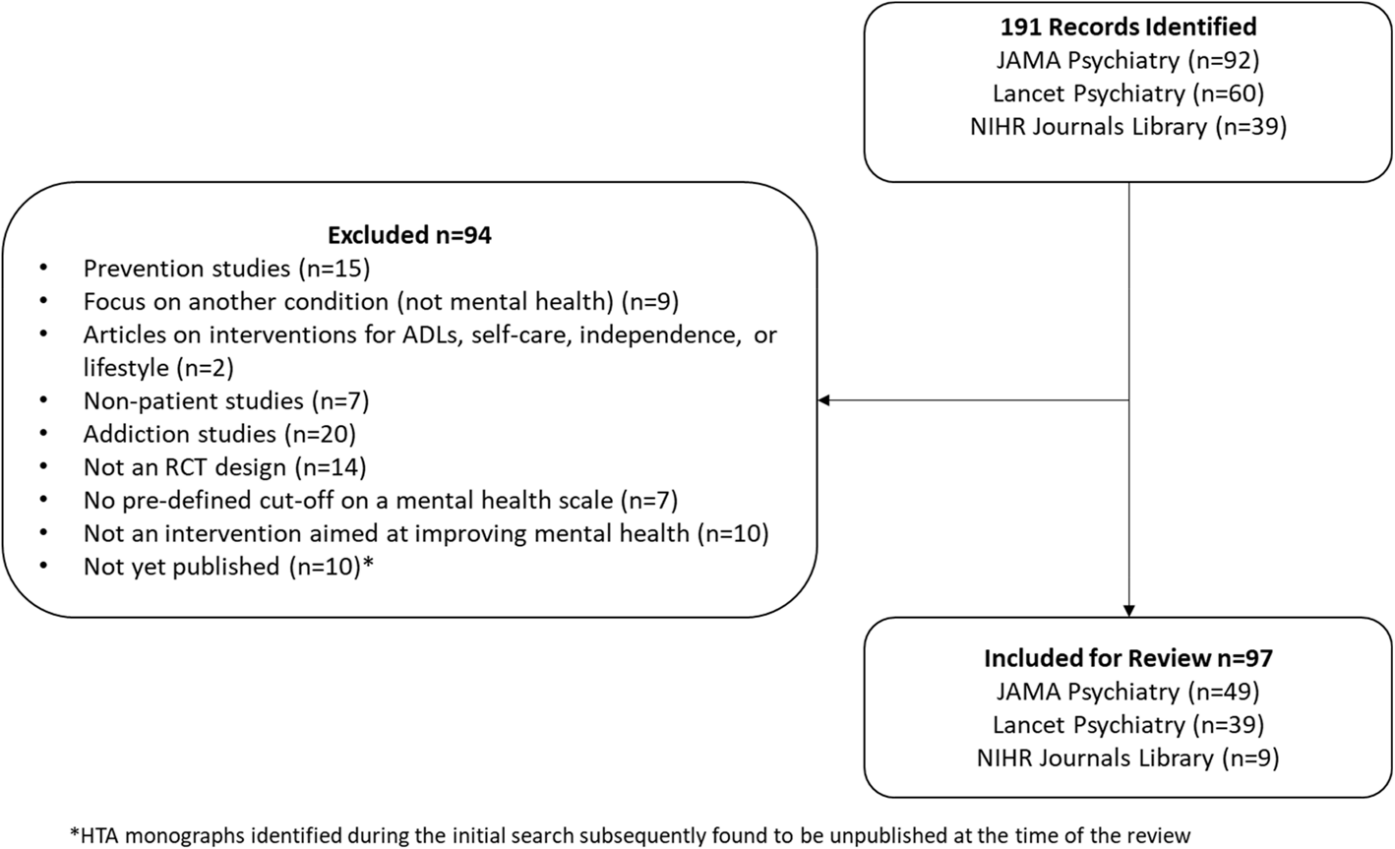
Scoping review flow diagram

A total of 191 records were identified from the search. After the screening of abstracts, 97 articles reporting either RCTs (90/97) or randomised feasibility or pilot studies (7/97) were included in the analysis (see Fig. 1). A detailed list of the articles included is provided in Additional file 1.

### Trial characteristics

Table 1 provides a breakdown of trial characteristics by recruitment strategy (offline, mixed or online). The vast majority of articles reported RCTs (90/97; 92.8%), most recruited through hospital settings (54/97; 55.7%) and were conducted in Europe or North America (81/97; 83.5%). The most common interventions used were psychological/behavioural (61/97; 62.9%); the most common conditions studied were mood disorders (35/97; 36.1%) and psychotic disorders (19/97; 19.6%). The sample included global research in mental health including a wide range of countries, settings, interventions and disorders.

**Table 1:** Baseline characteristics of included trials

	Recruitment strategy Total no. articles (*N* = 97)		
	Offline *n* = 66	Mixed *n* = 29	Online *n* = 2
Data source			
JAMA Psychiatry	28	19	2
Lancet Psychiatry	30	9	-
NIHR Journals Library	8	1	-
Trial design			
Randomised controlled trial	61	27	2
Randomised feasibility/pilot study	5	2	-
Type of intervention			
Psychological/behavioural	38	21	2
Drug	22	5	-
Mixed	3	2	-
Surgical/device	3	1	-
Setting			
Hospital (government or private)	38	16	-
Primary and community care (including care homes)	17	6	-
Academic institution, e.g. university	6	6	2
Mixed	5	1	-
Country/region			
Europe	35	9	2
North America	18	17	-
Australia	3	1	-
Africa	3	-	-
Asia	3	-	-
North America and Europe	2	1	-
Australia and Europe	1	-	-
North America, Europe and Asia	1	-	-
South America	-	1	-
Type of disorder			
Mood disorder (depression, anxiety)	25	10	-
Psychotic disorders (schizophrenia, persecutory delusions)	18	1	-
Personality/Behavioural disorders	8	1	-
Trauma-related disorders (PTSD)	5	4	-
Neurodevelopmental disorders (Autism, tics, ADHD)	2	4	-
Other^a^	3	1	-
Bipolar disorders	2	2	-
Phobias	-	2	1
Sleep disorders	1	1	1
Dementias	1	1	-
Obsessive Compulsive Disorder (OCD) and related disorders	-	2	-
Eating disorders	1	-	-

^a^Articles that included patients with a range of disorder types

### Recruitment strategy

**Fig. 2:**
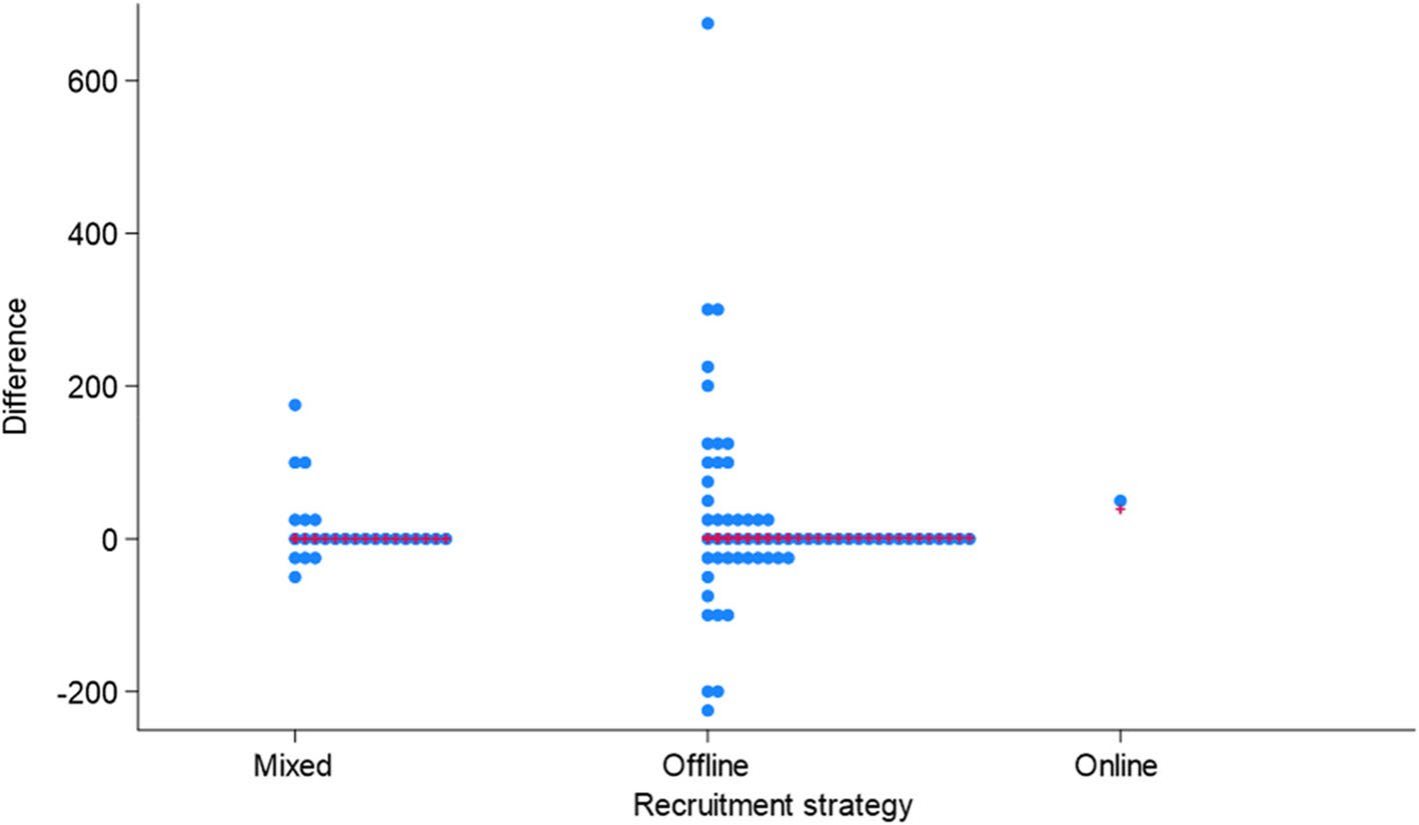
Dot plot of difference between observed and target sample size by recruitment strategy

**Fig. 3:**
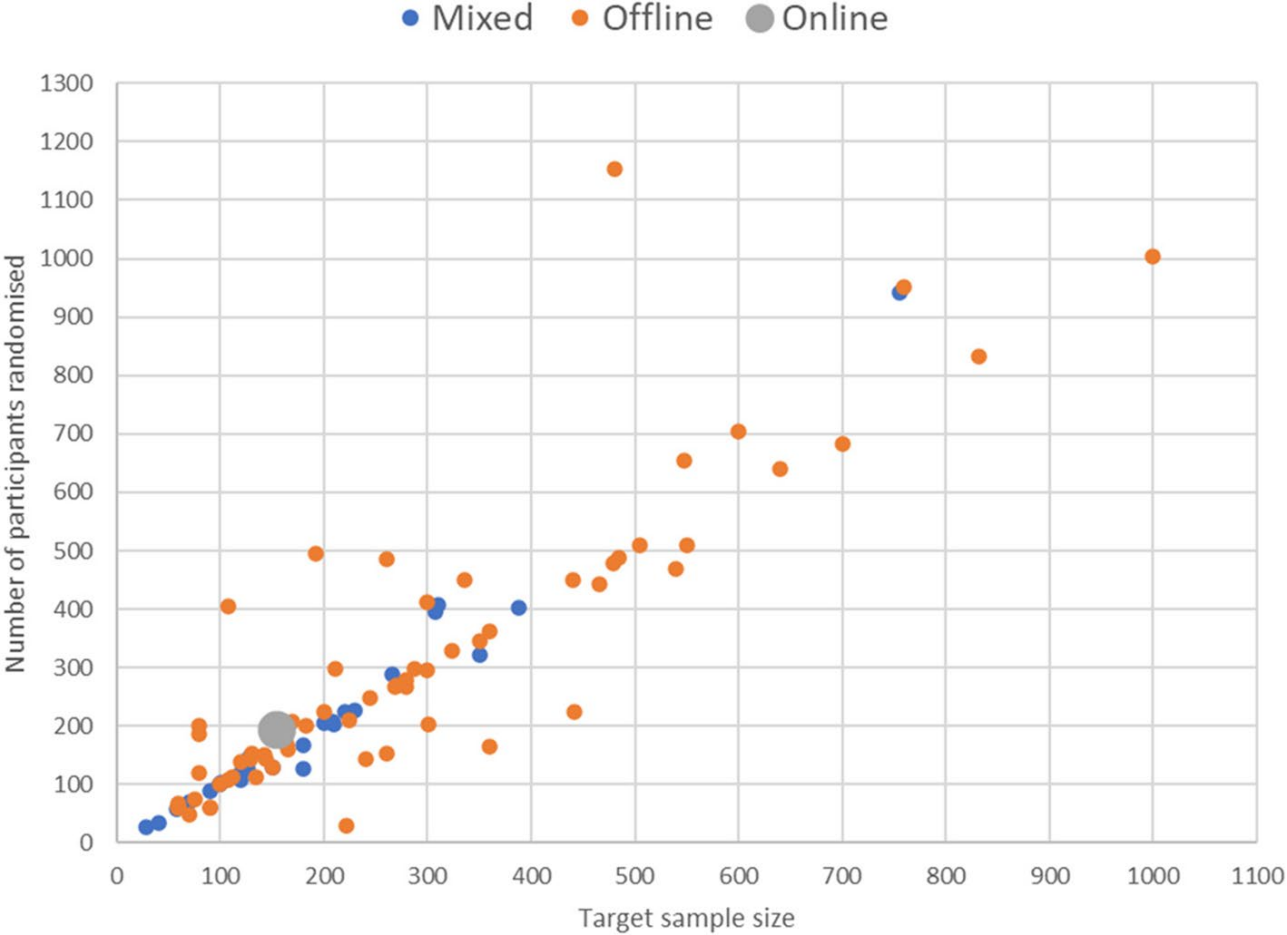
Scatterplot of the number of participants randomised versus target sample size by recruitment strategy

Our analysis showed no evidence of a relationship between the type of recruitment strategy (offline, mixed or online) and whether the trial recruited to target (68% offline, 62% mixed, 100% online; *p* = 0.67 see Table 2). Figures 2 and 3 both demonstrate that our data were dominated by offline trials. However, they highlight a suggestion that trials employing a mixed methods recruitment strategy may potentially be more efficient at recruiting than using offline methods. Only two trials were recruited using online methods; therefore, it was not possible to draw any comparative conclusions, although it is recognised that both trials recruited to target. Figure 2 shows quite neatly that over- and under-recruitment appears to be more prevalent in trials using an offline approach. The wider spread of the data is apparent visually but also evidenced through descriptive statistics (mixed approach—interquartile range (IQR) = 9.5 (− 5.5 to + 4); standard deviation 46.5; minimum − 53; maximum + 186/offline approach—IQR = 36.3 (− 13.3 to + 23); standard deviation 122.0; minimum − 218; maximum + 674). Likewise, Fig. 3 illustrates that the variability of the difference between observed and target recruitment appears to be smaller, on average, for a mixed recruitment strategy compared to an offline strategy. However, this possibility was not suspected a priori and is therefore hypothesis generating. This would clearly benefit from a more detailed exploration in a much larger number of trials using offline and online recruitment approaches.

**Table 2:** Recruited to target by recruitment strategy

	Recruitment strategy			
Recruited to target	Offline (%)	Mixed (%)	Online (%)	Total
N	21 (32)	10 (38)	0 (0)	31
Y	44 (68)	16 (62)	2 (100)	62
Total	65	26	2	93

% reported by column

Where a mixed recruitment strategy was used, although the range of methods were typically reported, e.g. website, twitter and mail out, they did not report a breakdown of how many participants were recruited via each method therefore this could not be further explored.

Table 3 shows recruitment strategy had little effect on whether a trial recruited to target for any of the included disorders. Offline was chosen more for psychotic disorders trials.

**Table 3:** Recruited to target by recruitment strategy and disorder

	Recruited to target^a^ (Y/N) by recruitment strategy						
	Offline		Mixed		Online		Total
Disorder	**N**	Y	**N**	Y	N	Y	
Emotional disorder (depression, anxiety)	8	16	6	4			34
Psychotic disorders (such as schizophrenia)	4	14		1			19
Personality/Behavioural disorders	4	4		1			9
Trauma-related disorders (e.g., post-traumatic stress disorder)	2	3	2	1			8
Neurodevelopmental disorders (Autism, Tics, ADHD)	1	1	1	2			5
Bipolar disorder	1	1	1	1			4
Others^b^		3		1			4
Sleep disorders	1			1		1	3
Dementias		1		1			2
OCD and related disorders				2			2
Phobia/fear of heights or spiders				1		1	2
Eating disorders		1					1
Grand Total	21	44	10	16	0	2	93

^a^Reported where data was available, four articles did not report whether target recruitment was achieved

^b^Articles that included patients with a range of disorder types

### Inclusivity

Table 4 reports the impact of recruitment strategy on inclusivity factors; gender, ethnicity and age of which the 97 articles were deemed representative in terms of reporting these factors. Nine articles were sex specific due to the nature of the condition and intervention, for example, postnatal depression.

**Table 4:** Recruitment by participant characteristics

	Count of participants Recruitment strategy			
	Mixed (%)	Offline (%)	Online (%)	Total (%)
Gender				
Male	2361 (42)	8353 (38)	446 (23)	11,160 (38)
Female	3178 (57)	13,242 (61)	1458 (77)	17,878(61)
Other	8 (1)	117 (1)	0 (0)	125 (1)
Total	5547	21,712	1904	29,163
Ethnicity^^^				
White	2391 (60)	10,360 (73)	1558 (91)	14,309 (72)
Black	235 (6)	2258 (16)	19 (1)	2512 (13)
Asian	125 (3)	357 (3)	0 (0)	482 (2)
Mixed ethnicity	16 (1)	2 (0)	0 (0)	18 (0)
Other	1216 (30)	1145 (8)	134 (8)	2495 (13)
Total	3983	14,122	1711	19,816
Agegroups				
Children/YP (0–25)	1105 (20)	4724 (22)	-	5829 (20)
Adult (16 +)	4043 (73)	15,953 (74)	1904 (100)	21,900 (75)
Older adult (60 +)	407 (7)	990 (4)	-	1397 (5)
Total	5555	21,667	1904	29,126

^^^Due to mixed reporting of ethnicity across papers, we used Gov.UK categorisations of ethnicity [30]. Where data did not fit one of these categorisations, they were included in the ‘other’ category

% reported for columns

In terms of overall numbers of participants recruited, there were small differences between the three recruitment strategies on diversity; 90/97 articles reported gender, but only a small number of articles (7/97; 7.2%) reported recruitment of other genders in addition to male/female, these predominantly recruited using offline strategies (6/97; 6.2%), 4 of which recruited to target. Slightly more male participants (42%) were recruited via mixed methods compared to offline methods (38%).

Of the trials that reported ethnicity (71/97; 73%), again offline methods were the largest recruiter in absolute numbers. It is worth noting this finding may be influenced by current service use across diverse ethnic groups. However, when combining ethnic groups (see Table 5), a mixed recruitment strategy appears to have the potential to improve inclusivity compared to using an offline recruitment strategy alone (40% vs 27%, respectively). The numbers for online recruitment are too small to draw any conclusions.

**Table 5:** Recruitment by non-white ethnicity combined

	Mixed (%)	Offline (%)	Online (%)	Total (%)
White	2391 (60)	10,360 (73)	1558 (91)	14,309 (72)
Combined ethnic groups	1592 (40)	3762 (27)	153 (9)	5507 (28)
Total	3983	14,122	1711	19,816

% reported for columns

Of articles that reported the age of participants (84/97; 87%), the absolute number of older adults (60 +) recruited was relatively small across all recruitment strategies; however, slightly more older adults were recruited via mixed methods compared to offline methods (7% vs 4%, respectively).

## Discussion

The present study was designed to review global evidence of recently published randomised controlled trials, feasibility or pilot studies in mental health, in order to assess the impact of online versus offline recruitment methods. The findings show no association between the use of any one recruitment strategy (offline, mixed or online) and whether the trial recruited to target or in relation to the size of the trial. A mixed recruitment strategy appears to recruit closer to target than an offline recruitment strategy. If this finding is replicated in a systematic review, it would mean that a mixed recruitment strategy is more efficient, reduces research waste and is more ethical (in terms of not unnecessarily exposing participants to the risk of adverse events, for example).

The quality and range of data reported on recruitment methods for the included trials were variable and impacted the analysis; this was a particular issue when analysing inclusivity data on age, gender and ethnicity. Evident in the data was that research participation in mental health trials in this sample (global high-quality published trials) was predominantly white, middle-aged and female, which reflects findings in previous research [31, 32]. Our study findings differ in that they suggest that using a mixed methods approach to participant recruitment may be more efficient than using offline methods alone and therefore may conversely help improve inclusivity and diversity, but it remains difficult to draw any solid conclusions from the evidence. Our finding is however in accordance with Dawson et al. [33] who recommend that the recruitment pathway does not limit and should enable participation. There was also support for a mixed methods approach to recruitment from our qualitative study of research staff and PPI opinions on recruitment strategies in mental health trials [27]. What is apparent is reporting of diversity including ethnicity remains inadequate despite efforts to mandate inclusion in the reporting of clinical trials, particularly in conditions that have known disparities in health by ethnicity [34].

We found no evidence to suggest that a particular recruitment method was more effective for any given age group, which is interesting when considering age-related stereotypes and technology acceptance and use [35]. Although the numbers for recruitment of older adults (60 +) were relatively small, approximately 30% were recruited via mixed methods, again suggesting that a range of methods may be preferable to meet the needs of a diverse population.

Only two trials were found solely to use online recruitment strategies which made direct comparison unfeasible. One reason for this could have been the timing of the review. It was conducted soon after the COVID-19 pandemic, and a high number of trials that were moved online due to the pandemic may not have yet completed. We would recommend an update of this review in 2 to 3 years’ time.

The results of the scoping review showed that using an offline strategy remains the predominant recruitment method in the trials that met our inclusion criteria. Possible explanations for this may result from our eligibility criteria that required a confirmed diagnosis which would indicate participants already accessing clinical services. It may also be explained in part by our qualitative data which highlighted the importance of relationship building and trust between the researcher/recruiter and the patient/participant [27]. This was considered by our participants to be particularly important in mental health trials, where sensitivity and confidentiality were deemed paramount both during recruitment conversations and to support continuing engagement [27]. We also found no evidence that any of the recruitment methods (offline, online or mixed) was more effective at recruiting participants with any particular disorder. Although for those mental disorders that may be deemed more severe, such as psychotic disorders, recruiting directly through clinical services was more common. This may be explained by the need to provide more clinical support including stringent safety monitoring for capacity to participate in research and/or the simple fact that these populations are likely being seen more regularly in a clinical setting, and so researchers have chosen to build their recruitment approach around an existing clinical pathway.

We identified mixed methods as a recruitment strategy in approximately a third of the included trials, with a small increase in the number of trials using this approach year on year (between 2017 and 2022) potentially demonstrating a trend towards mixed methods. This would also align with our recent qualitative study which found that using a range of recruitment methods was considered to help improve inclusivity by expanding opportunities for participation, particularly in mental illnesses which may be impacted by the prospect of direct contact, for example, anxiety or OCD [27]. Therefore, the selection of recruitment strategy and methods should be informed by the target participant population, so working closely with PPI, charities and clinical colleagues is essential to understand these individual needs [27].

## Limitations

Overall, we obtained data from well-known, reputable resources but this was not without its limitations. The scoping review included only three resources, the NIHR Journals Library and the top two mental health journals, Lancet Psychiatry and JAMA Psychiatry according to Google Scholar and citation metrics. Although through this selection process, we were able to identify high-quality research as evidenced by the journals’ peer review standards which is a strength, the included papers are limited to these sources. It is also worth noting that the quality standards of the journals may also have impacted on acceptance rates of articles. We may also have missed wider feasibility work in which aspects like recruitment strategies are tested and reported due to the requirements of the journals. This is borne out in that we only identified seven randomised feasibility and pilot trials for inclusion. In addition, only two studies were identified using solely online recruitment methods which impacted our analysis as we were unable to compare the recruitment strategies directly. High-quality articles from these journals would probably be published and led by a privileged group of researchers that may also lack diversity in the characteristics analysed as part of this study. However, we have stated this scoping review is a precursor study to inform the design of a full systematic review which would address these limitations. The timing of the systematic review will also allow us to include more trials that may have used online recruitment strategies but were delayed in completion due to the COVID-19 pandemic and therefore not published at the time of this review. The findings from this review will inform the search strategy and data extraction tools for the future systematic review. Lastly, comprehensive reporting of the trials was a limitation as there was no participant-level data reported on recruitment rates via the different methods used or whether these methods were adapted at any point during the trial recruitment phase. We were therefore unable to provide any direct evidence on which online methods may have been effective.

## Conclusions

The present study showed that there was no association between the use of any one recruitment strategy (offline or mixed) and whether the trial recruited to target in this cohort of trials. We found that a mixed methods approach may be more efficient than using offline methods alone and therefore may conversely help improve inclusivity and diversity, but this requires more evidence. We recommend that more consideration is given to the design, planning, and conduct of recruitment in mental health trials and for researchers to be more comprehensive in reporting the recruitment methods throughout the trial, to inform and improve recruitment strategies for future trials. This study, along with our recently published qualitative study, demonstrates that greater consideration should be given to online or mixed methods recruitment strategies that adopt a tailored approach, offering flexibility and choice to enable wider participation and improve inclusivity. The future systematic review will attempt to replicate these findings in a wider cohort of trials.

## Supplementary Information

Additional file 1.

Additional file 2.

## Data Availability

Data are available on reasonable request. The unpublished data used and/or analysed during the current study are available from the corresponding author on reasonable request.

## Abbreviations

COVID-19: The infectious disease caused by the SARS-CoV-2 virus
EDI: The Equality, Diversity and Inclusion
FDA: The Food and Drug Administration
NHS: The National Institute for Health Research
NIHR: The National Health Service
OCD: Obsessive–compulsive disorder
PPI: Patient and public involvement
RE-MIND: REcruitment in Mental health trials: broadening the ‘net’, opportunities for INclusivity through online methoDs
