# Supplementary material for: Strategies to improve recruitment in mental health clinical trials: a scoping review (RE-MIND study)
Source: Trials. 2024 Dec 18;25:832. doi: 10.1186/s13063-024-08665-x (PMC11654402; doi:10.1186/s13063-024-08665-x)
Supplement: Supplementary file 1 — Additional file 1. [file 13063_2024_8665_MOESM1_ESM.docx]

**Supplementary file 1: RE-MIND Scoping Review**

**1.1 Search strategy**

“randomi#ed controlled trial*” OR “randomi#ed trial*” OR “randomi#ed clinical trial*” AND “mental health” OR “mental illness*” or “mental disorder*” OR “psychiatr*” OR “psychol*” OR “Neurodevelopment*”

**1.2 Included trials and randomised feasibility studies in the evidence review**

JAMA - The Journal of the American Medical Association Psychiatry

LANCET - LANCET Psychiatry

NIHR – National Institute for Health and Care Excellence Journals Library

| **Source** | **Title** | **Authors** | **Year** | **Geographical region** | **Trial design** | **Sample size** | **Diagnosis/condition** | **Recruitment strategy** |
| --- | --- | --- | --- | --- | --- | --- | --- | --- |
| JAMA | Cognitive Behavioral Treatments for Anxiety in Children With Autism Spectrum Disorder: A Randomized Clinical Trial. JAMA Psychiatry. 2020;77(5):474–483. | Wood JJ, Kendall PC, Wood KS, et al | 2020 | North America | RCT | 167 | Emotional disorder (depression, anxiety) | Mixed |
| JAMA | Effects of Family-Focused Therapy vs Enhanced Usual Care for Symptomatic Youths at High Risk for Bipolar Disorder: A Randomized Clinical Trial. JAMA Psychiatry. 2020 May 1;77(5):455-463. | Miklowitz DJ, Schneck CD, Walshaw PD, et al. | 2020 | North America | RCT | 127 | Emotional disorder (depression, anxiety) | Mixed |
| JAMA | Effectiveness of Training Therapists to Deliver An Individualized Mental Health Intervention for Children With ASD in Publicly Funded Mental Health Services: A Cluster Randomized Clinical Trial. JAMA Psychiatry. 2019 Jun 1;76(6):574-583. | Brookman-Frazee L, Roesch S, Chlebowski C, et al. | 2019 | North America | RCT | 202 | Neurodevelopmental disorders (Autism, Tics, ADHD) | Offline |
| JAMA | Effectiveness of Transdiagnostic Cognitive-Behavioral Psychotherapy Compared With Management as Usual for Youth With Common Mental Health Problems: A Randomized Clinical Trial. JAMA Psychiatry. 2021 Mar 1;78(3):250-260. | Jeppesen P, Wolf RT, Nielsen SM, et al. | 2021 | Europe | RCT | 396 | Personality/Behavioural disorders | Mixed |
| JAMA | Effectiveness of Task-Shifted Trauma-Focused Cognitive Behavioral Therapy for Children Who Experienced Parental Death and Posttraumatic Stress in Kenya and Tanzania: A Randomized Clinical Trial. JAMA Psychiatry. 2020 May 1;77(5):464-473. | Dorsey S, Lucid L, Martin P, et al. | 2020 | Africa | RCT | 640 | Trauma-related disorders (such as post-traumatic stress disorder) | Offline |
| JAMA | Metabolic Effects of Antipsychotics on Adiposity and Insulin Sensitivity in Youths: A Randomized Clinical Trial. JAMA Psychiatry. 2018 Aug 1;75(8):788-796. | Nicol GE, Yingling MD, Flavin KS, et al. | 2018 | North America | RCT | 144 | Personality/Behavioural disorders | Offline |
| JAMA | Brief Behavioral Therapy for Pediatric Anxiety and Depression in Primary Care: A Randomized Clinical Trial. JAMA Psychiatry. 2017 Jun 1;74(6):571-578. | Weersing VR, Brent DA, Rozenman MS, et al. | 2017 | North America | RCT | 185 | Emotional disorder (depression, anxiety) | Offline |
| JAMA | Therapist-Guided Internet-Delivered Cognitive Behavioral Therapy vs Internet-Delivered Supportive Therapy for Children and Adolescents With Social Anxiety Disorder: A Randomized Clinical Trial. JAMA Psychiatry. 2021 Jul 1;78(7):705-713. | Nordh M, Wahlund T, Jolstedt M, et al. | 2021 | Europe | RCT | 103 | Emotional disorder (depression, anxiety) | Mixed |
| JAMA | Effect of Shamiri Layperson-Provided Intervention vs Study Skills Control Intervention for Depression and Anxiety Symptoms in Adolescents in Kenya: A Randomized Clinical Trial. JAMA Psychiatry. 2021 Aug 1;78(8):829-837. | Osborn TL, Venturo-Conerly KE, Arango G S, et al. | 2021 | Africa | RCT | 413 | Emotional disorder (depression, anxiety) | Offline |
| JAMA | Effect of Developmentally Adapted Cognitive Processing Therapy for Youth With Symptoms of Posttraumatic Stress Disorder After Childhood Sexual and Physical Abuse: A Randomized Clinical Trial. JAMA Psychiatry. 2019 May 1;76(5):484-491. | Rosner R, Rimane E, Frick U, et al. | 2019 | Europe | RCT | 88 | Trauma-related disorders (such as post-traumatic stress disorder) | Mixed |
| JAMA | Effect of 3 Forms of Early Intervention for Young People With Borderline Personality Disorder: The MOBY Randomized Clinical Trial. JAMA Psychiatry. 2022 Feb 1;79(2):109-119. | Chanen AM, Betts JK, Jackson H, et al. | 2022 | Australia | RCT | 139 | Personality/Behavioural disorders | Offline |
| JAMA | . Effect of Zuranolone vs Placebo in Postpartum Depression: A Randomized Clinical Trial. JAMA Psychiatry. 2021;78(9):951–959. | Deligiannidis KM, Meltzer-Brody S, Gunduz-Bruce H, et al | 2021 | North America | RCT | 153 | Emotional disorder (depression, anxiety) | Offline |
| JAMA | Maintenance of Wellness in Patients With Obsessive-Compulsive Disorder Who Discontinue Medication After Exposure/Response Prevention Augmentation: A Randomized Clinical Trial. JAMA Psychiatry. 2022 Mar 1;79(3):193-200. | Foa EB, Simpson HB, Gallagher T, et al. | 2022 | North America | RCT | 101 | OCD and related disorders | Mixed |
| JAMA | The Unified Protocol for Transdiagnostic Treatment of Emotional Disorders Compared With Diagnosis-Specific Protocols for Anxiety Disorders: A Randomized Clinical Trial. JAMA Psychiatry. 2017 Sep 1;74(9):875-884. | Barlow DH, Farchione TJ, Bullis JR, et al. | 2017 | North America | RCT | 223 | Emotional disorder (depression, anxiety) | Offline |
| JAMA | Effect of Online 1-Day Cognitive Behavioral Therapy-Based Workshops Plus Usual Care vs Usual Care Alone for Postpartum Depression: A Randomized Clinical Trial. JAMA Psychiatry. 2021 Nov 1;78(11):1200-1207. | Van Lieshout RJ, Layton H, Savoy CD, et al. | 2021 | North America | RCT | 403 | Emotional disorder (depression, anxiety) | Mixed |
| JAMA | Evaluation of Cognitive Behavioral Therapy vs Mindfulness Meditation in Brain Changes During Reappraisal and Acceptance Among Patients With Social Anxiety Disorder: A Randomized Clinical Trial. JAMA Psychiatry. 2021 Oct 1;78(10):1134-1142. | Goldin PR, Thurston M, Allende S, et al. | 2021 | North America | RCT | 108 | Emotional disorder (depression, anxiety) | Mixed |
| JAMA | Effectiveness of Predominantly Group Schema Therapy and Combined Individual and Group Schema Therapy for Borderline Personality Disorder: A Randomized Clinical Trial. JAMA Psychiatry. 2022;79(4):287–299. | Arntz A, Jacob GA, Lee CW, et al. | 2022 | Australia & Europe | RCT | 495 | Personality/Behavioural disorders | Offline |
| JAMA | Effect of Matching Therapists to Patients vs Assignment as Usual on Adult Psychotherapy Outcomes: A Randomized Clinical Trial. JAMA Psychiatry. 2021 Sep 1;78(9):960-969. | Constantino MJ, Boswell JF, Coyne AE, et al. | 2021 | North America | RCT | 288 | Others^a^ | Mixed |
| JAMA | Efficacy and Posttreatment Effects of Therapist-Delivered Cognitive Behavioral Therapy vs Supportive Psychotherapy for Adults With Body Dysmorphic Disorder: A Randomized Clinical Trial. JAMA Psychiatry. 2019 Apr 1;76(4):363-373. | Wilhelm S, Phillips KA, Greenberg JL, et al. | 2019 | North America | RCT | 120 | OCD and related disorders | Mixed |
| JAMA | Efficacy of Prolonged Exposure Therapy, Sertraline Hydrochloride, and Their Combination Among Combat Veterans With Posttraumatic Stress Disorder: A Randomized Clinical Trial. JAMA Psychiatry. 2019 Feb 1;76(2):117-126. | Rauch SAM, Kim HM, Powell C, et al. | 2019 | North America | RCT | 223 | Trauma-related disorders (such as post-traumatic stress disorder) | Offline |
| JAMA | Dialectical Behavior Therapy for Posttraumatic Stress Disorder (DBT-PTSD) Compared With Cognitive Processing Therapy (CPT) in Complex Presentations of PTSD in Women Survivors of Childhood Abuse: A Randomized Clinical Trial. JAMA Psychiatry. 2020 Dec 1;77(12):1235-1245. | Bohus M, Kleindienst N, Hahn C, et al. | 2020 | Europe | RCT | 200 | Trauma-related disorders (such as post-traumatic stress disorder) | Offline |
| JAMA | Pretreatment Rostral Anterior Cingulate Cortex Theta Activity in Relation to Symptom Improvement in Depression: A Randomized Clinical Trial. JAMA Psychiatry. 2018 Jun 1;75(6):547-554. | Pizzagalli DA, Webb CA, Dillon DG, et al. | 2018 | North America | RCT | 296 | Emotional disorder (depression, anxiety) | Offline |
| JAMA | Effect of Stellate Ganglion Block Treatment on Posttraumatic Stress Disorder Symptoms: A Randomized Clinical Trial. JAMA Psychiatry. 2020;77(2):130–138. | Rae Olmsted KL, Bartoszek M, Mulvaney S, et al. | 2020 | North America | RCT | 113 | Trauma-related disorders (such as post-traumatic stress disorder) | Offline |
| JAMA | Stratified Care vs Stepped Care for Depression: A Cluster Randomized Clinical Trial. JAMA Psychiatry. 2022;79(2):101–108. | Delgadillo J, Ali S, Fleck K, et al. | 2022 | Europe | RCT | 951 | Emotional disorder (depression, anxiety) | Offline |
| JAMA | Effect of Internet vs Face-to-Face Cognitive Behavior Therapy for Health Anxiety: A Randomized Noninferiority Clinical Trial. JAMA Psychiatry. 2020;77(9):915–924. | Axelsson E, Andersson E, Ljótsson B, et al. | 2020 | Europe | RCT | 204 | Emotional disorder (depression, anxiety) | Mixed |
| JAMA | Comparison of Teleintegrated Care and Telereferral Care for Treating Complex Psychiatric Disorders in Primary Care: A Pragmatic Randomized Comparative Effectiveness Trial. JAMA Psychiatry. 2021;78(11):1189–1199. | Fortney JC, Bauer AM, Cerimele JM, et al. | 2021 | North America | RCT | 1004 | Trauma-related disorders (such as post-traumatic stress disorder) | Offline |
| JAMA | Effect of Escalating and Deescalating Financial Incentives vs Usual Care to Improve Antidepressant Adherence: A Pilot Randomized Clinical Trial. JAMA Psychiatry. 2021 Feb 1;78(2):222-224. | Marcus SC, Reilly ME, Zentgraf K, Volpp KG, Olfson M. | 2021 | North America | Feasibility/  Pilot RCT | 120 | Emotional disorder (depression, anxiety) | Offline |
| JAMA | Effects of Psilocybin-Assisted Therapy on Major Depressive Disorder: A Randomized Clinical Trial. JAMA Psychiatry. 2021;78(5):481–489. | Davis AK, Barrett FS, May DG, et al. | 2021 | North America | RCT | 27 | Emotional disorder (depression, anxiety) | Mixed |
| JAMA | Effectiveness of Self-guided App-Based Virtual Reality Cognitive Behavior Therapy for Acrophobia: A Randomized Clinical Trial. JAMA Psychiatry. 2019 Jul 1;76(7):682-690. | Donker T, Cornelisz I, van Klaveren C, et al. | 2019 | Europe | RCT | 193 | Phobia/fear of heights or spiders | Online |
| JAMA | Coached Mobile App Platform for the Treatment of Depression and Anxiety Among Primary Care Patients: A Randomized Clinical Trial. JAMA Psychiatry. 2020 Sep 1;77(9):906-914. | Graham AK, Greene CJ, Kwasny MJ,et al. | 2020 | North America | RCT | 146 | Emotional disorder (depression, anxiety) | Mixed |
| JAMA | Efficacy and Safety of Lumateperone for Treatment of Schizophrenia: A Randomized Clinical Trial. JAMA Psychiatry. 2020;77(4):349–358. | Correll CU, Davis RE, Weingart M, et al. | 2020 | North America | RCT | 450 | Psychotic disorders (such as schizophrenia) | Offline |
| JAMA | Effects of SlowMo, a Blended Digital Therapy Targeting Reasoning, on Paranoia Among People With Psychosis: A Randomized Clinical Trial. JAMA Psychiatry. 2021;78(7):714–725. | Garety P, Ward T, Emsley R, et al. | 2021 | Europe | RCT | 361 | Psychotic disorders (such as schizophrenia) | Offline |
| JAMA | Effectiveness of Online Collaborative Care for Treating Mood and Anxiety Disorders in Primary Care: A Randomized Clinical Trial. JAMA Psychiatry. 2018 Jan 1;75(1):56-64. | Rollman BL, Herbeck Belnap B, Abebe KZ, et al. | 2018 | North America | RCT | 704 | Emotional disorder (depression, anxiety) | Offline |
| JAMA | Effect of Internet-Based Guided Self-help vs Individual Face-to-Face Treatment on Full or Subsyndromal Binge Eating Disorder in Overweight or Obese Patients: The INTERBED Randomized Clinical Trial. JAMA Psychiatry. 2017 Oct 1;74(10):987-995. | de Zwaan M, Herpertz S, Zipfel S, et al. | 2017 | Europe | RCT | 178 | Eating disorders | Offline |
| JAMA | A Brief Exposure-Based Treatment vs Cognitive Processing Therapy for Posttraumatic Stress Disorder: A Randomized Noninferiority Clinical Trial. JAMA Psychiatry. 2018 Mar 1;75(3):233-239. | Sloan DM, Marx BP, Lee DJ, Resick PA. | 2018 | North America | RCT | 126 | Trauma-related disorders (such as post-traumatic stress disorder) | Mixed |
| JAMA | Efficacy and Safety of Intranasal Esketamine Adjunctive to Oral Antidepressant Therapy in Treatment-Resistant Depression: A Randomized Clinical Trial. JAMA Psychiatry. 2018;75(2):139–148. | Daly EJ, Singh JB, Fedgchin M, et al. | 2018 | North America | RCT | 67 | Emotional disorder (depression, anxiety) | Offline |
| JAMA | Effect of Disorder-Specific vs Nonspecific Psychotherapy for Chronic Depression: A Randomized Clinical Trial. JAMA Psychiatry. 2017 Mar 1;74(3):233-242. | Schramm E, Kriston L, Zobel I, et al. | 2017 | Europe | RCT | 268 | Emotional disorder (depression, anxiety) | Offline |
| JAMA | Effectiveness of Sequential Psychological and Medication Therapies for Insomnia Disorder: A Randomized Clinical Trial. JAMA Psychiatry. 2020 Nov 1;77(11):1107-1115. | Morin CM, Edinger JD, Beaulieu-Bonneau S, et al. | 2020 | North America | RCT | 211 | Sleep disorders | Offline |
| JAMA | Efficacy and Safety of Transcranial Direct Current Stimulation as an Add-on Treatment for Bipolar Depression: A Randomized Clinical Trial. JAMA Psychiatry. 2018 Feb 1;75(2):158-166. | Sampaio-Junior B, Tortella G, Borrione L, et al. | 2018 | South America | RCT | 59 | Bipolar disorder | Mixed |
| JAMA | Effect of Digital Cognitive Behavioral Therapy for Insomnia on Health, Psychological Well-being, and Sleep-Related Quality of Life: A Randomized Clinical Trial. JAMA Psychiatry. 2019 Jan 1;76(1):21-30. | Espie CA, Emsley R, Kyle SD, et al. | 2019 | Europe | RCT | 1711 | Sleep disorders | Online |
| JAMA | Effect of Repetitive Transcranial Magnetic Stimulation on Treatment-Resistant Major Depression in US Veterans: A Randomized Clinical Trial. JAMA Psychiatry. 2018 Sep 1;75(9):884-893. | Yesavage JA, Fairchild JK, Mi Z, et al. | 2018 | North America | RCT | 164 | Emotional disorder (depression, anxiety) | Offline |
| JAMA | Effect of Exercise, Escitalopram, or Placebo on Anxiety in Patients With Coronary Heart Disease: The Understanding the Benefits of Exercise and Escitalopram in Anxious Patients With Coronary Heart Disease (UNWIND) Randomized Clinical Trial. JAMA Psychiatry. 2021 Nov 1;78(11):1270-1278. | Blumenthal JA, Smith PJ, Jiang W, et al. | 2021 | North America | RCT | 128 | Emotional disorder (depression, anxiety) | Mixed |
| JAMA | Effectiveness and Safety of Dementia Care Management in Primary Care: A Randomized Clinical Trial. JAMA Psychiatry. 2017 Oct 1;74(10):996-1004. | Thyrian JR, Hertel J, Wucherer D, et al. | 2017 | Europe | RCT | 407 | Dementias | Mixed |
| JAMA | Efficacy of Yoga vs Cognitive Behavioral Therapy vs Stress Education for the Treatment of Generalized Anxiety Disorder: A Randomized Clinical Trial. JAMA Psychiatry. 2021 Jan 1;78(1):13-20. | Simon NM, Hofmann SG, Rosenfield D, et al. | 2021 | North America | RCT | 226 | Emotional disorder (depression, anxiety) | Mixed |
| JAMA | Efficacy of Digital Cognitive Behavioral Therapy for the Treatment of Insomnia Symptoms Among Pregnant Women: A Randomized Clinical Trial. JAMA Psychiatry. 2020 May 1;77(5):484-492. | Felder JN, Epel ES, Neuhaus J, Krystal AD, Prather AA. | 2020 | North America | RCT | 208 | Sleep disorders | Mixed |
| JAMA | Efficacy of Esketamine Nasal Spray Plus Oral Antidepressant Treatment for Relapse Prevention in Patients With Treatment-Resistant Depression: A Randomized Clinical Trial. JAMA Psychiatry. 2019 Sep 1;76(9):893-903. | Daly EJ, Trivedi MH, Janik A, et al. | 2019 | North America & Europe | RCT | 297 | Emotional disorder (depression, anxiety) | Offline |
| JAMA | Efficacy of Adjunctive Infliximab vs Placebo in the Treatment of Adults With Bipolar I/II Depression: A Randomized Clinical Trial. JAMA Psychiatry. 2019 Aug 1;76(8):783-790. | McIntyre RS, Subramaniapillai M, Lee Y, et al. | 2019 | North America | RCT | 60 | Bipolar disorder | Offline |
| JAMA | Efficacy and Tolerability of Adjunctive Intravenous Sodium Nitroprusside Treatment for Outpatients With Schizophrenia: A Randomized Clinical Trial. JAMA Psychiatry. 2019 Jul 1;76(7):691-699. | Brown HE, Freudenreich O, Fan X, et al. | 2019 | North America | RCT | 60 | Psychotic disorders (such as schizophrenia) | Offline |
| JAMA | Effect of Adjunctive Estradiol on Schizophrenia Among Women of Childbearing Age: A Randomized Clinical Trial. JAMA Psychiatry. 2019 Oct 1;76(10):1009-1017. | Weiser M, Levi L, Zamora D, et al. | 2019 | Europe | RCT | 200 | Psychotic disorders (such as schizophrenia) | Offline |
| LANCET | Therapist-supported online remote behavioural intervention for tics in children and adolescents in England (ORBIT): a multicentre, parallel group, single-blind, randomised controlled trial. Lancet Psychiatry. 2021 Oct;8(10):871-882. | Hollis C, Hall CL, Jones R, et al. | 2021 | Europe | RCT | 224 | Neurodevelopmental disorders (Autism, Tics, ADHD) | Mixed |
| LANCET | Effectiveness of systemic family therapy versus treatment as usual for young people after self-harm: a pragmatic, phase 3, multicentre, randomised controlled trial. Lancet Psychiatry. 2018 Mar;5(3):203-216. | Cottrell DJ, Wright-Hughes A, Collinson M, et al. | 2018 | Europe | RCT | 832 | Personality/Behavioural disorders | Offline |
| LANCET | Cognitive behavioural therapy and short-term psychoanalytical psychotherapy versus a brief psychosocial intervention in adolescents with unipolar major depressive disorder (IMPACT): A multicentre, pragmatic, observer-blind, randomised controlled superiority trial. The Lancet Psychiatry, 2017 4(2), 109–119. | Goodyer, I. M, Reynolds, S., Barrett, B., et al. | 2017 | Europe | RCT | 470 | Emotional disorder (depression, anxiety) | Offline |
| LANCET | Quetiapine extended release versus aripiprazole in children and adolescents with first-episode psychosis: the multicentre, double-blind, randomised tolerability and efficacy of antipsychotics (TEA) trial. Lancet Psychiatry. 2017 Aug;4(8):605-618. | Pagsberg AK, Jeppesen P, Klauber DG, et al. | 2017 | Europe | RCT | 113 | Psychotic disorders (such as schizophrenia) | Offline |
| LANCET | Comparison of effectiveness and cost-effectiveness of an intensive community supported discharge service versus treatment as usual for adolescents with psychiatric emergencies: a randomised controlled trial. Lancet Psychiatry. 2018 Jun;5(6):477-485. | Ougrin D, Corrigall R, Poole J, et al. | 2018 | Europe | RCT | 108 | Psychotic disorders (such as schizophrenia) | Offline |
| LANCET | Antipsychotic medication versus psychological intervention versus a combination of both in adolescents with first-episode psychosis (MAPS): a multicentre, three-arm, randomised controlled pilot and feasibility study. Lancet Psychiatry. 2020 Sep;7(9):788-800. | Morrison AP, Pyle M, Maughan D, et al | 2020 | Europe | Feasibility/  Pilot RCT | 61 | Psychotic disorders (such as schizophrenia) | Offline |
| LANCET | The addition of fluoxetine to cognitive behavioural therapy for youth depression (YoDA-C): a randomised, double-blind, placebo-controlled, multicentre clinical trial. Lancet Psychiatry. 2019 Sep;6(9):735-744. | Davey CG, Chanen AM, Hetrick SE, et al. | 2019 | Australia | RCT | 153 | Emotional disorder (depression, anxiety) | Offline |
| LANCET | Brain-based mediation of non-conscious reduction of phobic avoidance in young women during functional MRI: a randomised controlled experiment. Lancet Psychiatry. 2020 Nov;7(11):971-981. | Siegel P, Wang Z, Murray L, et al. | 2020 | North America | RCT | 82 | Phobia/fear of heights or spiders | Mixed |
| LANCET | Antipsychotic drugs versus cognitive behavioural therapy versus a combination of both in people with psychosis: a randomised controlled pilot and feasibility study. Lancet Psychiatry. 2018 May;5(5):411-423. Epub 2018 Apr 5. Erratum in: Lancet Psychiatry. 2019 Jul;6(7):e16. | Morrison AP, Law H, Carter L, et al. | 2019 | Europe | Feasibility/  Pilot RCT | 75 | Psychotic disorders (such as schizophrenia) | Offline |
| LANCET | The benefit of minocycline on negative symptoms of schizophrenia in patients with recent-onset psychosis (BeneMin): a randomised, double-blind, placebo-controlled trial. Lancet Psychiatry. 2018 Nov;5(11):885-894. | Deakin B, Suckling J, Barnes TRE, et al. | 2018 | Europe | RCT | 207 | Psychotic disorders (such as schizophrenia) | Offline |
| LANCET | Effectiveness of a peer-delivered, psychosocial intervention on maternal depression and child development at 3 years postnatal: a cluster randomised trial in Pakistan. Lancet Psychiatry. 2020 Sep;7(9):775-787. | Maselko J, Sikander S, Turner EL, et al. | 2020 | Asia | RCT | 1154 | Emotional disorder (depression, anxiety) | Offline |
| LANCET | Efficacy and safety of balovaptan for socialisation and communication difficulties in autistic adults in North America and Europe: a phase 3, randomised, placebo-controlled trial. Lancet Psychiatry. 2022 Mar;9(3):199-210. Epub 2022 Feb 10. | Jacob S, Veenstra-VanderWeele J, Murphy D, et al. | 2022 | North America & Europe | RCT | 322 | Neurodevelopmental disorders (Autism, Tics, ADHD) | Mixed |
| LANCET | Amisulpride, aripiprazole, and olanzapine in patients with schizophrenia-spectrum disorders (BeSt InTro): a pragmatic, rater-blind, semi-randomised trial. Lancet Psychiatry. 2020 Nov;7(11):945-954. | Johnsen E, Kroken RA, Løberg EM, et al. | 2020 | Europe | RCT | 144 | Psychotic disorders (such as schizophrenia) | Offline |
| LANCET | Efficacy and safety of the novel glycine transporter inhibitor BI 425809 once daily in patients with schizophrenia: a double-blind, randomised, placebo-controlled phase 2 study. Lancet Psychiatry. 2021 Mar;8(3):191-201. | Fleischhacker WW, Podhorna J, Gröschl M, et al. | 2021 | North America, Canada, Europe & Asia | RCT | 509 | Psychotic disorders (such as schizophrenia) | Offline |
| LANCET | Automated virtual reality therapy to treat agoraphobic avoidance and distress in patients with psychosis (gameChange): a multicentre, parallel-group, single-blind, randomised, controlled trial in England with mediation and moderation analyses. Lancet Psychiatry. 2022 May;9(5):375-388. | Freeman D, Lambe S, Kabir T, et al. | 2022 | Europe | RCT | 346 | Psychotic disorders (such as schizophrenia) | Offline |
| LANCET | Person-centred experiential therapy versus cognitive behavioural therapy delivered in the English Improving Access to Psychological Therapies service for the treatment of moderate or severe depression (PRaCTICED): a pragmatic, randomised, non-inferiority trial. Lancet Psychiatry. 2021 Jun;8(6):487-499. | Barkham M, Saxon D, Hardy GE, et al. | 2021 | Europe | RCT | 510 | Emotional disorder (depression, anxiety) | Offline |
| LANCET | Clinical efficacy of a Decision Support Tool (Link-me) to guide intensity of mental health care in primary practice: a pragmatic stratified randomised controlled trial. Lancet Psychiatry. 2021 Mar;8(3):202-214. | Fletcher S, Spittal MJ, Chondros P, et al. | 2021 | Australia | RCT | 1671 | Emotional disorder (depression, anxiety) | Offline |
| LANCET | The clinical effectiveness of sertraline in primary care and the role of depression severity and duration (PANDA): a pragmatic, double-blind, placebo-controlled randomised trial. Lancet Psychiatry. 2019 Nov;6(11):903-914. | Lewis G, Duffy L, Ades A, et al. | 2019 | Europe | RCT | 655 | Emotional disorder (depression, anxiety) | Offline |
| LANCET | Comparison of behavioural activation with guided self-help for treatment of depression in adults with intellectual disabilities: a randomised controlled trial. Lancet Psychiatry. 2017 Dec;4(12):909-919. | Jahoda A, Hastings R, Hatton C, et al. | 2017 | Europe | RCT | 161 | Emotional disorder (depression, anxiety) | Offline |
| LANCET | Comparison of a theoretically driven cognitive therapy (the Feeling Safe Programme) with befriending for the treatment of persistent persecutory delusions: A parallel, single-blind, randomised controlled trial. The Lancet Psychiatry,2021 8(8), 696–707. | Freeman, D., Emsley, R., Diamond, R., et al. | 2021 | Europe | RCT | 130 | Psychotic disorders (such as schizophrenia) | Offline |
| LANCET | 3,4-methylenedioxymethamphetamine (MDMA)-assisted psychotherapy for post-traumatic stress disorder in military veterans, firefighters, and police officers: a randomised, double-blind, dose-response, phase 2 clinical trial. Lancet Psychiatry. 2018 Jun;5(6):486-497. | Mithoefer MC, Mithoefer AT, Feduccia AA, et al. | 2018 | North America | RCT | 28 | Trauma-related disorders (such as post-traumatic stress disorder) | Mixed |
| LANCET | Cognitive behavioural therapy in clozapine-resistant schizophrenia (FOCUS): An assessor-blinded, randomised controlled trial. The Lancet Psychiatry, 2018 5(8), 633–643. | Morrison, A. P., Pyle, M., Gumley, A., et al. | 2018 | Europe | RCT | 487 | Psychotic disorders (such as schizophrenia) | Offline |
| LANCET | AVATAR therapy for auditory verbal hallucinations in people with psychosis: a single-blind, randomised controlled trial. Lancet Psychiatry. 2018 Jan;5(1):31-40. | Craig TK, Rus-Calafell M, Ward T, et al. | 2018 | Europe | RCT | 150 | Psychotic disorders (such as schizophrenia) | Offline |
| LANCET | Automated psychological therapy using immersive virtual reality for treatment of fear of heights: a single-blind, parallel-group, randomised controlled trial. Lancet Psychiatry. 2018 Aug;5(8):625-632. | Freeman D, Haselton P, Freeman J, et al. | 2018 | Europe | RCT | 100 | Phobia/fear of heights or spiders | Mixed |
| LANCET | Long versus short pulse width subcallosal cingulate stimulation for treatment-resistant depression: a randomised, double-blind, crossover trial. Lancet Psychiatry. 2020 Jan;7(1):29-40. | Ramasubbu R, Clark DL, Golding S, et al. | 2020 | North America | RCT | 22 | Emotional disorder (depression, anxiety) | Offline |
| LANCET | Efficacy and cost-effectiveness of task-shared care for people with severe mental disorders in Ethiopia (TaSCS): a single-blind, randomised, controlled, phase 3 non-inferiority trial. Lancet Psychiatry. 2022 Jan;9(1):59-71. | Hanlon C, Medhin G, Dewey ME, et al. | 2022 | Africa | RCT | 329 | Others^a^ | Offline |
| LANCET | Deutetrabenazine for treatment of involuntary movements in patients with tardive dyskinesia (AIM-TD): a double-blind, randomised, placebo-controlled, phase 3 trial. Lancet Psychiatry. 2017 Aug;4(8):595-604. | Anderson KE, Stamler D, Davis MD, et al. | 2017 | North America & Europe | RCT | 298 | Others^a^ | Offline |
| LANCET | Antipsychotic treatment of very late-onset schizophrenia-like psychosis (ATLAS): a randomised, controlled, double-blind trial. Lancet Psychiatry. 2018 Jul;5(7):553-563. | Howard R, Cort E, Bradley R, et al. | 2018 | Europe | RCT | 101 | Psychotic disorders (such as schizophrenia) | Offline |
| LANCET | Clinical and cost-effectiveness of the Managing Agitation and Raising Quality of Life (MARQUE) intervention for agitation in people with dementia in care homes: a single-blind, cluster-randomised controlled trial. Lancet Psychiatry. 2019 Apr;6(4):293-304. | Livingston G, Barber J, Marston L, et al. | 2019 | Europe | RCT | 404 | Dementias | Offline |
| LANCET | Multisystemic therapy versus management as usual in the treatment of adolescent antisocial behaviour (START): 5-year follow-up of a pragmatic, randomised, controlled, superiority trial. Lancet Psychiatry. 2020 May;7(5):420-430. | Fonagy P, Butler S, Cottrell D, et al. | 2020 | Europe | RCT | 684 | Personality/Behavioural disorders | Offline |
| LANCET | Mitigating the effect of persistent postnatal depression on child outcomes through an intervention to treat depression and improve parenting: A randomised controlled trial. The Lancet Psychiatry, 2018 5(2), 134–144. | Stein, A., Netsi, E., Lawrence, P. J., et al. | 2018 | Europe | RCT | 144 | Emotional disorder (depression, anxiety) | Offline |
| LANCET | Minocycline and celecoxib as adjunctive treatments for bipolar depression: a multicentre, factorial design randomised controlled trial. Lancet Psychiatry. 2020 Jun;7(6):515-527. | Husain MI, Chaudhry IB, Khoso AB, et al. | 2020 | Asia | RCT | 266 | Bipolar disorder | Offline |
| LANCET | Lurasidone versus treatment as usual for cognitive impairment in euthymic patients with bipolar I disorder: a randomised, open-label, pilot study. Lancet Psychiatry. 2017 Mar;4(3):208-217. | Yatham LN, Mackala S, Basivireddy J, et al. | 2017 | North America | Feasibility/  Pilot RCT | 34 | Bipolar disorder | Mixed |
| LANCET | Neurofeedback, sham neurofeedback, and cognitive-behavioural group therapy in adults with attention-deficit hyperactivity disorder: a triple-blind, randomised, controlled trial. Lancet Psychiatry. 2017 Sep;4(9):673-684. | Schönenberg M, Wiedemann E, Schneidt A, et al. | 2017 | Europe | RCT | 118 | Neurodevelopmental disorders (Autism, Tics, ADHD) | Mixed |
| LANCET | Financial incentives for improving adherence to maintenance treatment in patients with psychotic disorders (Money for Medication): a multicentre, open-label, randomised controlled trial. Lancet Psychiatry. 2017 Mar;4(3):199-207. | Noordraven EL, Wierdsma AI, Blanken P, et al. | 2017 | Europe | RCT | 169 | Psychotic disorders (such as schizophrenia) | Offline |
| LANCET | Non-trauma-focused meditation versus exposure therapy in veterans with post-traumatic stress disorder: a randomised controlled trial. Lancet Psychiatry. 2018 Dec;5(12):975-986. | Nidich S, Mills PJ, Rainforth M, et al. | 2018 | North America | RCT | 203 | Trauma-related disorders (such as post-traumatic stress disorder) | Mixed |
| LANCET | REFOCUS-PULSAR recovery-oriented practice training in specialist mental health care: a stepped-wedge cluster randomised controlled trial. Lancet Psychiatry. 2019 Feb;6(2):103-114. | Meadows G, Brophy L, Shawyer F, et al. | 2019 | Australia | RCT | 942 | Psychotic disorders (such as schizophrenia) | Mixed |
| LANCET | Combined social communication therapy at home and in education for young autistic children in England (PACT-G): a parallel, single-blind, randomised controlled trial. Lancet Psychiatry. 2022 Apr;9(4):307-320. d | Green J, Leadbitter K, Ellis C, et al. | 2022 | Europe | RCT | 249 | Neurodevelopmental disorders (Autism, Tics, ADHD) | Offline |
| LANCET | Delivering the Thinking Healthy Programme for perinatal depression through volunteer peers: a cluster randomised controlled trial in Pakistan. Lancet Psychiatry. 2019 Feb;6(2):128-139. | Sikander S, Ahmad I, Atif N, et al. | 2019 | Asia | RCT | 280 | Emotional disorder (depression, anxiety) | Offline |
| NIHR | An intervention for parents with severe personality difficulties whose children have mental health problems: a feasibility RCT. Health Technol Assess. 2020 Mar;24(14):1-188. | Day C, Briskman J, Crawford MJ, et al. | 2020 | Europe | Feasibility/  Pilot RCT | 48 | Personality/Behavioural disorders | Offline |
| NIHR | Guided self-help for depression in autistic adults: the ADEPT feasibility RCT. Health Technol Assess. 2019 Dec;23(68):1-94. | Russell A, Gaunt D, Cooper K, et al. | 2019 | Europe | Feasibility/  Pilot RCT | 70 | Neurodevelopmental disorders (Autism, Tics, ADHD) | Mixed |
| NIHR | Cognitive-behaviour therapy for health anxiety in medical patients (CHAMP): a randomised controlled trial with outcomes to 5 years. Health Technol Assess. 2017 Sep;21(50):1-58. | Tyrer P, Salkovskis P, Tyrer H, et al. | 2017 | Europe | RCT | 444 | Emotional disorder (depression, anxiety) | Offline |
| NIHR | Group cognitive rehabilitation to reduce the psychological impact of multiple sclerosis on quality of life: the CRAMMS RCT. Health Technol Assess 2020;24(4) | Lincoln NB, Bradshaw LE, Constantinescu CS et al. | 2020 | Europe | RCT | 449 | Others^a^ | Offline |
| NIHR | Antidepressant medication to prevent depression relapse in primary care: the ANTLER RCT. Health Technol Assess. 2021 Nov;25(69):1-62. | Duffy L, Clarke CS, Lewis G, et al. | 2021 | Europe | RCT | 478 | Emotional disorder (depression, anxiety) | Offline |
| NIHR | CollAborative care for Screen-Positive EldeRs with major depression (CASPER plus): a multicentred randomised controlled trial of clinical effectiveness and cost-effectiveness. Health Technol Assess. 2017 Nov;21(67):1-252. | Bosanquet K, Adamson J, Atherton K, et al. | 2017 | Europe | RCT | 485 | Emotional disorder (depression, anxiety) | Offline |
| NIHR | Psychological intervention, antipsychotic medication or a combined treatment for adolescents with a first episode of psychosis: the MAPS feasibility three-arm RCT. Health Technol Assess. 2021 Jan;25(4):1-124. | Morrison AP, Pyle M, Byrne R, et al. | 2021 | Europe | Feasibility/  Pilot RCT | 61 | Psychotic disorders (such as schizophrenia) | Offline |
| NIHR | Social recovery therapy for young people with emerging severe mental illness: the Prodigy RCT. Health Technol Assess. 2021 Nov;25(70):1-98. | Fowler D, Berry C, Hodgekins J, et al. | 2021 | Europe | RCT | 270 | Personality/Behavioural disorders | Offline |
| NIHR | The Clinical Effectiveness and Cost Effectiveness of Clozapine for Inpatients with Borderline Personality Disorder: A Randomised Controlled Trial. Lessons learned report https://www.fundingawards.nihr.ac.uk/award/16/157/02 | Crawford M, Lesson V, Evans R, Hoare Z et al | 2021 | Europe | RCT | 29 | Personality/Behavioural disorders | Offline |

^a^ – articles that included patients with a range of disorder types
